# Middle East respiratory syndrome coronavirus infection: virus-host cell interactions and implications on pathogenesis

**DOI:** 10.1186/s12985-015-0446-6

**Published:** 2015-12-22

**Authors:** Jie Zhou, Hin Chu, Jasper Fuk-Woo Chan, Kwok-Yung Yuen

**Affiliations:** State Key Laboratory of Emerging Infectious Diseases, The University of Hong Kong, Hong Kong Special Administrative Region, China; Department of Microbiology, The University of Hong Kong, Queen Mary Hospital, 102 Pokfulam Road, Hong Kong Special Administrative Region, China; Research Centre of Infection and Immunology, The University of Hong Kong, Hong Kong Special Administrative Region, China; Carol Yu Centre for Infection, The University of Hong Kong, Hong Kong Special Administrative Region, China

## Abstract

Middle-East Respiratory Syndrome coronavirus (MERS-CoV) was identified to cause severe respiratory infection in humans since 2012. The continuing MERS epidemic with a case-fatality of more than 30 % poses a major threat to public health worldwide. Currently, the pathogenesis of human MERS-CoV infection remains poorly understood. We reviewed experimental findings from human primary cells and ex vivo human lung tissues, as well as those from animal studies, so as to understand the pathogenesis and high case-fatality of MERS. Human respiratory epithelial cells are highly susceptible to MERS-CoV and can support productive viral replication. However, the induction of antiviral cytokines and proinflammatory cytokines/chemokines are substantially dampened in the infected epithelial cells, due to the antagonistic mechanisms evolved by the virus. MERS-CoV can readily infect and robustly replicate in human macrophages and dendritic cells, triggering the aberrant production of proinflammatory cytokines/chemokines. MERS-CoV can also effectively infect human primary T cells and induce massive apoptosis in these cells. Although data from clinical, in vitro and ex vivo studies suggested the potential for virus dissemination, extrapulmonary involvement in MERS patients has not been ascertained due to the lack of autopsy study. In MERS-CoV permissive animal models, although viral RNA can be detected from multiple organs of the affected animals, the brain of human DPP4-transgenic mouse was the only extrapulmonary organ from which the infectious virus can be recovered. More research findings on the pathogenesis of MERS and the tissue tropisms of MERS-CoV may help to improve the treatment and infection control of MERS.

## Background

### General overview of MERS and MERS-CoV

In September 2012, a novel lineage C betacoronavirus was identified as the etiological agent to cause severe respiratory infection in humans in the Middle East [1]. The novel virus was formally named as Middle East respiratory syndrome coronavirus (MERS-CoV) by the Coronavirus Study Group of International Committee on Taxonomy of Viruses in 2013 [2]. As of 23 September 2015, MERS-CoV has caused 1570 infection cases and 555 deaths in over 20 countries worldwide, with a high case-fatality of more than 30 % [3]. The continuing MERS epidemic in the Middle East is believed to be related to the failure to control the zoonotic sources, most probably the dromedary camels, which results in ongoing camel-to-human transmission [4–9]. While all primary cases of MERS have been epidemiologically linked to the Middle East, numerous clusters in the community and healthcare settings have also been reported. The largest healthcare-associated outbreak occurred in the Republic of Korea in 2015, in which 186 cases including 36 deaths occurred after the index patient returned from the Middle East [10]. The high case-fatality rate of MERS and the capability of MERS-CoV to cause outbreaks in healthcare facilities pose significant threat to public health worldwide.

Unlike most other human-pathogenic coronaviruses, which mainly cause self-limiting upper respiratory tract infections, MERS-CoV is capable of causing severe disease with lower respiratory tract involvement and extrapulmonary manifestations [11, 12]. Patients with severe MERS often present with pneumonic symptoms including fever, cough and dyspnea, with some progressing to respiratory failure and acute respiratory distress syndrome [13, 14]. Extrapulmonary manifestations such as renal failure, hepatic dysfunction, and diarrhea are not uncommon [13, 15]. Additionally, deranged coagulation profile and hematological changes may also be observed in severe MERS [15]. The infectious virus could be recovered exclusively from patients’ respiratory tract, whilst viral RNA could be detected extrapulmonary specimens such as blood, urine and stool by nucleic acid amplification tests [16, 17]. These clinical and laboratory findings are suggestive of possibly disseminated viral infection.

MERS bears some resemblance to severe acute respiratory syndrome (SARS) in terms  of clinical manifestation. The etiological agent of SARS is a lineage B betacoronavirus, SARS coronavirus (SARS-CoV). In 2002–2003, SARS-CoV infected more than 8000 patients with a case-fatality rate of 9.6 %, affecting over 30 countries worldwide [18–20]. However, unlike SARS, no autopsy study has been conducted in deceased MERS patients. Therefore, our understanding of the pathogenesis of MERS remains largely elusive. In this review, we summarize the current knowledge on MERS-CoV cellular tropisms and the virus-host interaction observed in in vitro human cells, ex vivo human tissues, and their implications on the pathogenesis of MERS. The relevant findings in MERS-CoV infected experimental animals are also reviewed, aiming to recapitulate the human MERS disease and to better the understanding of pathogenesis of human MERS-CoV infection.

## Cellular tropism of MERS-CoV in respiratory system and innate immune response upon infection

The primary infection site of MERS is human respiratory tract. It was demonstrated that MERS-CoV could effectively infect and robustly replicate in the human airway epithelium [21, 22]. Ex vivo tissues from human respiratory tract were also examined for the cellular tropism of MERS-CoV [23, 24]. It was shown that MERS-CoV infected non-ciliated bronchial epithelial cells, bronchiolar epithelial cells, alveolar epithelial cells and endothelial cells of pulmonary vessels [23]. Additionally, upon MERS-CoV infection in ex vivo lung tissues, the uninfected cells underwent massive apoptosis with extensive caspase 3 activation, implicating that paracrine mechanism may contribute to the induction of apoptosis [23]. Another study in human ex vivo lung tissue echoed the discovery of MERS-CoV’s productive infection and replication, as well as the occurrence of infection-induced apoptosis in human lung tissue [24]. Moreover, it was shown that MERS-CoV infection in ex vivo lung tissues displayed the comparable extent and kinetics to the infection of highly pathogenic avian H5N1 influenza virus in lung tissues of the same donors, indicating the high infectivity of MERS-CoV in human respiratory epithelium [24]. However, in the latter study, MERS-CoV could also infect ciliated bronchial epithelial cells while these cells were not susceptible to the virus in the former study [23, 24]. Notably, the studies in human ex vivo airway tissues invariably revealed that pulmonary endothelial cells were highly susceptible to MERS-CoV [23, 24], suggesting that MERS-CoV infection in respiratory tract could potentially develop into a systematic or disseminated infection. Taken together, human respiratory epithelium is highly permissive to MERS-CoV. The effective infection results in robust viral propagation and massive induction of apoptosis. These studies provided pathological basis of the major pulmonary features of MERS i.e., pneumonia and acute lung injury.

As the first line of host defense, human epithelial cells are stimulated to produce antiviral and proinflammatory cytokines and chemokines to eliminate the invading pathogens. Based on the studies in human primary respiratory epithelial cells, respiratory epithelial cell lines and ex vivo human lung tissue, there has been a consensus recognition that the induction of antiviral interferons (i.e. type I and III IFNs) was basically dampened, although type I and type III IFNs treatment can effectively inhibit the MERS-CoV replication [21–23]. Additionally, MERS-CoV infection failed to elicit strong proinflammatory cytokines response in human primary respiratory epithelial cells and ex vivo respiratory tissues [21, 23, 24]. The respiratory epithelial cell line Calu-3 cells were used to study the early and late phase of innate immune response after MERS-CoV infection. It was demonstrated that the proinflammatory cytokines/chemokines such as IL-1β, IL-8 and IL-6 exhibited a delayed but marked induction upon MERS-CoV infection [25]. Based on these observations, antagonistic mechanisms of MERS-CoV to attenuate innate immune response have been extensively sought. A number of MERS-CoV proteins, including the papain-like protease, membrane protein, and accessory proteins 4a, 4b and 5, have been identified to suppress the interferon production [26–29]. Among them, MERS-CoV 4a was shown to suppress PACT-induced activation of RIG-I and MDA5 and circumvent the innate immune response [27]. Additionally, MERS-CoV papain-like protease exhibited the deubiquitinating and deISGylating activities and suppressed the innate immune response [29]. Collectively, MERS-CoV may have evolved multiple antagonistic mechanisms to dampen or attenuate the host defense, which contributed to the high pathogenicity in humans.

Consistent with the responsiveness of MERS-CoV to interferon treatment in vitro, combinational treatment of IFN-α2b and ribavirin reduced virus replication and improved clinical outcome in MERS-CoV infected rhesus macaques [30]. It was further demonstrated that IFN-β1b treatment showed a better outcome in the virus-inoculated common marmosets [31].

## The human immune cells involved in MERS-CoV infection

While the cellular tropism of SARS-CoV in human respiratory tract resembles that of MERS-CoV [21, 22], a number of immune cells displayed distinct susceptibility to MERS-CoV and SARS-CoV.

### Macrophages

The novel MERS-CoV has a broad tissue and cellular tropism including human monocytic cell line, THP-1 [32]. A subsequent study focused on human macrophages and demonstrated that MERS-CoV could efficiently infect and replicate in human monocyte-derived macrophages (MDMs) [33]. Notably, a 2–4-log increase in viral RNA was consistently detected in MERS-CoV infected MDMs derived from different donors within 48 h post infection. In addition, MERS-CoV significantly induced the expression of proinflammatory and chemotactic cytokines and chemokines, including IP-10/CXCL-10, MCP-1/CCL-2, MIP-1α/CCL-3, RANTES/CCL-5, IL-8, and IL-12, in the infected human macrophages [33]. In the meantime, MERS-CoV triggered the upregulation of MHC class I-, MHC class II-, and costimulation-related genes in MDMs. Furthermore, by utilizing ex vivo organ culture, the authors demonstrated that the alveolar macrophages in human lung tissues were susceptible to MERS-CoV [33].

The SARS-CoV can infect human macrophages. However, viral replication of SARS-CoV in human macrophages was abortive and no infectious virus particles were produced [34–37]. Despite an abortive infection, the SARS-CoV infection in human macrophages induced the expression of a number of proinflammatory chemokines including IP-10/CXCL10 and MCP-1/CCL2. On the other hand, the induction of IFN-α and IFN-β, which are important antiviral cytokines and key components of innate immunity, was largely absent [35, 37].

### Dendritic cells

Dendritic cells are essential sentinels of the immune system, which detect invading pathogens and bridge the innate immune system with the adaptive immune system. Infection of human monocyte-derived dendritic cells (mDCs) by MERS-CoV was productive as evidenced by the progressive increase in viral antigen expression, increase in viral RNA, and increase in virus titer in the culture media of the infected cells [38]. Intriguingly, in the same study, the authors reported that MERS-CoV induced no IFN-β and marginal IFN-α expression in infected dendritic cells. On the other hand, MERS-CoV triggered substantial expression of IFN-γ, IL-12, IP-10/CXCL-10 and RANTES/CCL-5, which was significantly higher than that of SARS-CoV-infected dendritic cells. In addition, the surface expression of MHC class II and costimulatory molecule was disturbed in MERS-CoV-infected dendritic cells, which might contribute to the immune dysregulation during MERS-CoV infection [38]. Recently, Scheuplein et al. reported the infection of plasmacytoid dendritic cells by MERS-CoV. Remarkably, the author concluded that although MERS-CoV infection in plasmacytoid dendritic cells was abortive, the infection induced the production of large amounts of type I and type III IFNs exclusively in these cells [39].

In the case of SARS-CoV, a number of studies demonstrated that human dendritic cells were susceptible to SARS-CoV but were unable to support viral replication [35, 36, 40]. Nonetheless, SARS-CoV infection led to the phenotypic and functional maturation of dendritic cells, with regard to MHC class II and costimulatory molecule expression, T cell-stimulatory capacity, and cytokine/chemokine production including TNF-α, MIP-1α/CCL-3, RANTES/CCL-5, IP-10/CXCL-10, and MCP-1/CCL-2 [35, 40]. Importantly, Zhao et al. demonstrated the potential role of dendritic cells in controlling the pathogenesis of SARS-CoV in a mouse study. In particular, the authors showed that severe outcome of SARS-CoV infection correlated with the slow kinetics of virus clearance and delayed activation of respiratory dendritic cells [41].

Collectively, in contrast to the inability to elicit the production of proinflammatory cytokines in human respiratory epithelial cells, MERS-CoV and SARS-CoV are able to stimulate the induction of proinflammatory cytokines/chemokines in human macrophages and dendritic cells. However, the question arises whether the triggered innate immune response is beneficial or detrimental in MERS-CoV and SARS-CoV infected patients since the host immune response may act as a two-edged sword at different stages of disease [42]. Therefore, further studies are warranted in experimental animals and patients to elucidate how the virus-host interaction in these immune cells affect the pathogenesis of human coronavirus infections.

### T lymphocytes

Although lymphopenia is commonly observed in SARS and MERS patients, the exact cause of lymphopenia currently remains unknown. It has been postulated that SARS-CoV may directly infect T cells and lead to T cell depletion [43]. However, alternative models for lymphopenia upon SARS-CoV infection have also been proposed, including sequestration of lymphocytes within the inflamed tissues, cytokine-induced cell death, as well as suppression of hematopoietic progenitor cells in bone marrow or thymus [37]. As for the infection of MERS-CoV, a recent study demonstrated that the infection of human DPP4-transduced and T cell-deficient mice with MERS-CoV resulted in the persistence of MERS-CoV in the lungs while the virus was cleared in control mice and B cell-deficient mice. These findings hinted that T cells might play critical roles in controlling the pathogenesis of MERS-CoV [44]. Subsequently, a study published earlier this year directly addressed the issue of MERS-CoV infection in T cells. The authors demonstrated that T cells from human peripheral blood mononuclear cells, human lymphoid tissues, and the spleen of common marmosets were highly susceptible to MERS-CoV. Furthermore, MERS-CoV induced substantial apoptosis in the infected T cells that involved the activation of the intrinsic and extrinsic caspase-dependent apoptosis pathways [45]. The results suggested that the unusual capacity of MERS-CoV to infect T cells and induce apoptosis might contribute to the high pathogenicity of the virus.

The role of T cells in controlling the pathogenesis of SARS-CoV infection remains incompletely understood. In a mouse study, it was observed that depletion of CD4+ T cells resulted in enhanced interstitial pneumonitis and delayed clearance of SARS-CoV from the lung tissues, which was associated with reduced production of neutralizing antibody and cytokines as well as reduced pulmonary recruitment of lymphocytes [46].

## Possible involvement of extrapulmonary organs upon MERS-CoV infection

MERS-CoV viral RNA could be detected in blood, urine and stool specimens of some MERS patients, suggesting that the virus dissemination may occur [17]. We and others have shown that endothelial cells of blood vessel in human ex vivo lung tissues were permissive to MERS-CoV [23, 33], which may provide the pathological basis of the potential virus dissemination. In addition, it has been demonstrated that MERS-CoV can infect human monocyte-derived dendritic cells and cause productive viral replication [38]. Human primary T cells are also readily susceptible to MERS-CoV [45]. Dendritic cells and T cells are migrating cells in the human body. Therefore, the MERS-CoV infected dendritic cells and T cells may allow the virus to disseminate systemically beyond the respiratory tract. Collectively, extrapulmonary organs and tissues are very likely to be involved in MERS-CoV infection in vivo. However, so far, no human autopsy study has been documented. To address the possible extrapulmonary involvement, we have to seek evidence from MERS-CoV infected experimental animals although none of these animals can fully recapitulate the human MERS disease.

### MERS-CoV infection and pathogenesis in non-human primate model and small animal models

The receptor for MERS-CoV was identified to be an exopeptidase, dipeptidyl peptidase 4 (DPP4) [47]. The role of DPP4 as the main determinant in the host tropism of MERS-CoV has been elucidated in several studies [48–50]. Commonly used laboratory animal species such as Syrian hamster, mice and ferrets are not susceptible to MERS-CoV since DPP4 orthologs of these animal species are unable to bind MERS-CoV spike protein and mediate virus entry [49, 51, 52]. The MERS-CoV inoculation in rabbits displayed an asymptomatic infection. Neither significant histopathological change nor clinical symptom was observed in these rabbits although the virus could be detected from lung tissues [53]. Camels are susceptible to the MERS-CoV isolated from human. Although the infected camels can shed large amounts of virus from the upper respiratory tract, the disease signs were mild [54]. Among all experimental animals tested for the development of MERS animal models, rhesus macaques developed a mild to moderate respiratory infection [55, 56] whereas common marmosets displayed a moderate to severe respiratory disease after inoculation using a combination routes of intranasal, intra-tracheal, oral and ocular [57]. In marmosets, the MERS-CoV infection was suggested to be a disseminated infection since viral RNA was detectable in nearly all tested tissues in all infected animals, including blood, kidney, intestine, liver and spleen etc. However, except for the samples from the respiratory tract, isolation of infectious virus in other organs was not successful. Interestingly, the susceptibility of macrophages to MERS-CoV as evidenced in the in vitro and ex vivo studies was verified in alveolar macrophages of MERS-CoV infected marmoset [57].

The first MERS mouse model was generated by prior transduction of adenoviral vector expressing human DPP4 (hDPP4). These mice displayed a transient viral pneumonia which resolved within 1–2 weeks after infection [44]. Several lines of human DPP4 transgenic mice have been subsequently reported. MERS-CoV infection and replication were invariably evidenced in these hDPP4 transgenic mice [58–60]. However, disease sign and pathology in these mice differed, which appeared to depend on the promoters controlling the expression of hDPP4 gene. Pascal et al. replaced the mouse DPP4 ORF with human DPP4 so that the knocked-in hDPP4 is under the control of the endogenous mouse DPP4 promoter [60]. The authors believed that this strategy may render hDPP4 to be expressed in a physiologically-relevant context. After virus inoculation in these mice, MERS-CoV robustly replicated in the mouse lung. However, the inoculated mice did not exhibit disease signs, without any extrapulmonary involvement. On the other hand, two lines of hDPP4 transgenic mice which had the transgene under the control of chicken β-actin promoter [58] and cytokeratin 18 promoter [59] respectively, were highly permissive to MERS-CoV infection. The mice developed progressive pneumonia with fatal outcome after intranasal inoculation. The infectious virus can constantly and exclusively be recovered from lung and brain tissues [58, 59]. Similar to the marmoset study, while viral RNA can also be detected from extrapulmonary organs including the heart, spleen and intestines, virus isolation from these organs was unsuccessful. The hDPP4 transgenic mouse with cytokeratin 18 (CK18) promoter was generated in parallel with another transgenic mouse line with surfactant protein C (SPC) promoter. SPC promoter confers transgene expression in bronchiolar and alveolar epithelia while CK18 promoter can drive a more universal transgene expression in epithelia of liver, kidney, gastrointestinal tract and some cells in the nervous system, apart from respiratory tract. The hDPP4 mice with CK18 promoter developed lethal infection after intranasal inoculation of MERS-CoV. In contrast, the same inoculation in the hDPP4 mice with SPC promoter caused no mortality or body weight loss [59]. Therefore, the morbidity and mortality in human DPP4 transgenic mice may correlate to the tissue/cellular distribution and/or the expression intensity of the transgene. Notably, a common discovery among these MERS-CoV susceptible animals was that the gene expression of antiviral cytokines, proinflammatory cytokines and chemokines was elevated [55, 57, 58]. Collectively, the tissue tropisms of MERS-CoV in human hosts have not been fully elucidated although there has been accumulating evidence of possible extrapulmonary involvement in MERS patients. Undoubtedly, hDPP4 transgenic mouse studies are conducive for the development of antivirals or vaccines against MERS-CoV. However, in terms of implication for the pathogenesis in human MERS, findings from these hDPP4 transgenic mice must be interpreted with great caution. An in-depth investigation of the tissue tropisms of MERS-CoV in human hosts will facilitate our understanding towards the transmission route and pathogenesis of MERS.

The MERS-CoV mouse models have been utilized to test the efficacy of antiviral drug, neutralization antibody and vaccine. A Venezuelan equine encephalitis replication particle expressing MERS-CoV spike protein (VRP-S) was demonstrated to substantially reduce the virus titer in lung tissues of the immunized hDPP4 transduced mouse model [44]. The same group subsequent examined the VRP-S in a more permissive hDPP4 transgenic mouse model [59]. The VRP-S immunized hDPP4 transgenic mice were completely protected from lethal infection. Pretreatment with serum of the mouse immunized with VRP-S can also be protected from fatal infection [59]. Two fully human neutralization antibodies binding to distinct epitopes of MERS spike protein, which were generated using the mouse expressing human antibody, displayed the pre- and post-exposure protection efficacy from MERS-CoV infection in hDPP4 transgenic mice [61].

## Conclusions

The pathogenesis of human MERS-CoV infection remains poorly understood. Human respiratory epithelium is highly susceptible to MERS-CoV and can support productive viral replication. However, MERS-CoV has evolved multiple antagonistic mechanisms to attenuate the induction of antiviral and proinflammatory cytokines in the affected epithelial cells. Additionally, MERS-CoV can readily infect and robustly replicate in human macrophages and dendritic cells, which elicits the aberrant production of proinflammatory cytokines/chemokines. MERS-CoV can also effectively infect human primary T cells and induce massive apoptosis in these cells. Although clinical presentations as well as in vitro and ex vivo studies implicated the potential virus dissemination upon MERS-CoV infection, the extrapulmonary involvement has not been ascertained. In the MERS-CoV permissive animal models, viral RNA can be detected from multiple organs of the infected animals. However, recovery of the infectious virus was unsuccessful in most extrapulmonary organs. More studies are warranted to further characterize the tissue tropisms of MERS-CoV for the better understanding towards the pathogenesis of MERS.
